# Aqueous Liquid-Liquid Phase Separation of Natural and Synthetic Polyguanidiniums

**DOI:** 10.3390/polym11040649

**Published:** 2019-04-09

**Authors:** Leland J. Prather, G. Mahika Weerasekare, Monika Sima, Colette Quinn, Russell J. Stewart

**Affiliations:** 1 Department of Biomedical Engineering, University of Utah, Salt Lake City, UT 84112, USA; l.jack.prather@gmail.com (L.J.P.); mahikaw@yahoo.com (G.M.W.); ms57@utah.edu (M.S.); 2 TA Instruments, 890 W 410 N St, Lindon, UT 84042, USA; cquinn@tainstruments.com

**Keywords:** liquid-liquid phase separation, complex coacervation, Gdm^+^ like-charge pairing, protamine, salmine, guanidinium, polyguanidinium

## Abstract

Protamines are natural polyguanidiniums, arginine(R)-rich proteins involved in the compaction of chromatin during vertebrate spermatogenesis. Salmine, a protamine isolated from salmon sperm, contains 65 mol% R residues, with positively charged guanidino (Gdm^+^) sidechains, and no other amino acids with ionizable or aromatic sidechains. Salmine sulfate solutions undergo liquid-liquid phase separation (LLPS) with a concentration-dependent upper critical solution temperature (UCST). The condensed liquid phase comprises 50 wt % water and >600 mg·mL^−1^ salmine with a constant 1:2 ratio of sulfate (SO_4_^2−^) to Gdm^+^. Isothermal titration calorimetry, titrating Na_2_SO_4_ into salmine chloride above and below the UCST, allowed isolation of exothermic sulfate binding to salmine chloride from subsequent endothermic condensation and exothermic phase separation events. Synthetic random polyacrylate analogs of salmine, with 3-guanidinopropyl sidechains, displayed similar counterion dependent phase behavior, demonstrating that the LLPS of polyguanidiniums does not depend upon subunit sequence or polymer backbone chirality, and was due entirely to Gdm^+^ sidechain interactions. The results provide experimental evidence for like-charge pairing of Gdm^+^ sidechains, and an experimental approach for further characterizing these interactions.

## 1. Introduction

Complex coacervation, a type of liquid-liquid phase separation (LLPS), occurs when attractive interactions between solvated macromolecules are strong enough to condense the macromolecules into a separate phase, yet are weak and dynamic enough that the condensed phase forms a fluid network, rather than a solid precipitate or ionic hydrogel [1]. In suitable solution conditions, all types of non-covalent interactions between macromolecules, including ion pairing, H-bonding, π–π, π–cation, and metal-ligand interactions, can promote LLPS by complex coacervation [2,3,4]. Cellular LLPS creates transient, phase-defined cytoplasmic compartments, also called membraneless organelles, in response to external stimuli [5,6,7,8,9]. A growing list of proteins known to condense in the cytoplasm and in vitro, contain polycationic arginine-rich motifs [10,11,12,13,14,15], pointing to the importance of arginines (R) in the intermolecular associations that lead to cellular LLPS. Enzymatic methylation of Rs diminishes the LLPS of R-rich Ddx4 [11] and FUS [8,15,16], which suggests cellular LLPS may be regulated, at least in part, through enzymatic R methylation, further highlighting the role of Rs in cellular LLPS. Disruption of normal cellular LLPS, or gain-of-function mutations that cause abnormal cellular LLPS, lead to pathology, examples of which include neurodegenerative diseases [17,18,19]. Therefore, better understanding of the molecular mechanisms of R-mediated LLPS may provide insights into fundamental cell biology, and have important implications for health and disease.

Protamines are small, R-rich proteins that replace histones during spermatogenesis to compact chromatin into the sperm heads of vertebrates [20]. In vitro associative LLPS of purified protamine sulfate was reported in the early 20th century when Kossel described the “condensation of an oily precipitate” from a warm aqueous solution of protamine sulfate as it cooled [21]. Salmine is a protamine isolated from salmon sperm comprising 32 amino acid residues, of which 21 are R [22]. Salmine contains no other charged or aromatic amino acids. As such, salmine is a convenient and readily available R-rich protein with which to investigate the role of R-R interactions in associative LLPS in the absence of other electrostatic or π–cation interactions. Here, we describe initial physicochemical characterization of the in vitro LLPS of salmine. The temperature, concentration, and counter-ion dependence, as well as the thermodynamics of the condensation reaction are described. To show that the condensation is mediated through association of R-Gdm^+^ sidechains, the phase behavior of a series of poly(3-guanidinopropyl methacrylamide-co-acrylamide) random copolymers—synthetic salmine analogs—were partially characterized for comparison.

## 2. Materials and Methods

### 2.1. Salmine Sulfate and Salmine Chloride

USP grade salmine sulfate, obtained from MP Biomedicals, Inc., Santa Ana, CA, USA (CAS: 9009-65-8). The M_m_ of the salmine-AI GenBank sequence (accession number X07511.1, (MPRRRRSSSR PVRRRRRPRVSRRRRRRGGRRRR), 4381 g·mol^−1^, was used to estimate the concentrations of positively charged guanidino (Gdm^+^) sidechains (21/32 amino acids) and molar ratios of Gdm^+^ to sulfate (SO_4_^2−^) in salmine solutions. For example, the [Gdm^+^] in a 50 mg mL^−1^ solution was estimated to be 194 mM from the sequence, and the [SO_4_^2−^] as 99 mM from elemental analysis of the as-received salmine sulfate. The SO_4_^2−^:Gdm^+^ molar ratio was 0.51. To convert salmine sulfate to the chloride form, it was dissolved in ultrapure deionized water at 10 mg mL^−1^, then passed through an ion exchange column, Amberlite IRA-400 HCl (Aldrich, CAS: 60177-39-1, St. Louis, MO, USA). The exchanged protamine was dialyzed to remove excess salt, then lyophilized. The phosphate and acetate forms of salmine were prepared by similar ion exchange processes on Amberlite resins.

### 2.2. Coacervate Characterization

The salmine concentrations in the coacervate phase and the supernatant were determined by quantitative nuclear magnetic resonance (NMR) using *t*-butanol as an internal standard [23]. In a typical experiment, a 100 mg·mL^−1^ solution of salmine sulfate at pH 7 was heated to 65 °C, mixed with a 1 M solution of Na_2_SO_4_ at 65 °C, then diluted to a final concentration of 50 mg mL^−1^ salmine sulfate (194 mM Gdm^+^). The solution was then equilibrated at a given temperature overnight. The two phases were separated at that temperature and lyophilized. The dried coacervate and the dried supernatant phase were then dissolved separately in D_2_O and 1.5*M* HCl in D_2_O, once dissolved, *t*-butanol in D_2_O was added to the solutions. The integral of *t*-butanol signal (1.03 ppm) was set to 1.00. The integral for arginine δCH_2_ (3.01 ppm) was used to calculate the percent protamine in the coacervate and supernatant. A similar method substituting ethylene glycol (3.40 ppm) for *t*-butanol was used to calculate the amount of poly(3-guanidinopropyl methacrylamide-co-acrylamide) in the coacervate and supernatant as the *t*-butanol proton signals overlap with the backbone proton signals of the polymer.

Coacervate yield and water content was determined gravimetrically. Salmine sulfate and salmine chloride were dissolved at a concentration of 50 mg·mL^−1^ in varying concentrations of Na_2_SO_4_, or NaCl solutions. The solutions were equilibrated overnight at 8 °C. After separating the supernatant and coacervate, the mass and volume of each phase was determined before lyophilization. 

The wet weight compared to the dry weight was used to determine the wt % water in the coacervate phase. The coacervate yield was determined from the dry weight and wet volume. ICP-OES was used to determine the molar ratio of S to Gdm^+^ in the coacervate.

### 2.3. Phase Transition Temperature Determination by Turbidity

Turbidity at 600 nm was determined in a Lamda Bio 20 UV/Vis Spectrometer (PerkinElmer, Waltham, MA, USA). Neither salmine nor poly(3-guanidinopropyl methacrylamide-co-acrylamide) solutions absorb light at 600 nm. The temperature within the spectrometer was controlled using a Peltier system (Perkin Elmer, PTP-6, Waltham, MA, USA) with a precision of 0.1 °C. Each sample was initially heated to 60 °C, and then lowered in 1 °C increments until the transmission fell to 70% of its initial value, which was recorded as the liquid-liquid phase separation (LLPS) upper critical solution temperature (UCST).

### 2.4. Isothermal Titration Calorimetry (ITC)

An Affinity ITC LV isothermal titration calorimeter (TA Instruments, New Castle, DE, USA) was used to determine the enthalpic and entropic changes resulting from the titration of salmine chloride (10 mg·mL^−1^, 41.4 mM R) with a solution of Na_2_SO_4_ (400 mM) above the phase transition temperature (25 °C) and below the phase transition temperature (10 °C). Prior to the first injection, the system was equilibrated to a medium setting of 0.3 µW·hr^−1^ and 0.03 µW standard error. Thirty-five one μL injections of 400 mM Na_2_SO_4_ were injected into an active cell volume of 185 μL of salmine chloride. Each injection was delivered over 2 s with 200 s between successive injections. The injection syringe was attached to a paddle, and the solution was stirred at 100 rpm during the assay. Data is plotted in Js^−1^, and exothermic events are downward peaks in the thermograms.

A thermogram of 400 Mm of Na_2_SO_4_ injected into a NaCl solution (41.4 mM) without the salmine produced constant heat, which served as a negative control, and the background heat of dilution. The [NaCl] was equivalent to the amount of Cl^−^ present in the salmine chloride sample to avoid ionic strength effects. salmine ITC thermograms were corrected for this background heat of dilution, 443.6 μJ and 816.6 μJ for the 25 and 10 °C experiments, respectively. Data acquisition and analyses were performed using NanoAnalyze^®^ (TA Instruments, New Castle, DE, USA) which was supplied with the instrument. The one-site model, provided with the software, was used to fit binding isotherms above the UCST (25 °C). The one-site and multiple-sites model were both used to fit binding isotherms below the UCST (10 °C). Thermodynamic parameters, including the binding constant (K_a_), binding stoichiometry (n), change in enthalpy (ΔH), and change in entropy (ΔS) were calculated by iterative curve fitting of the binding isotherms. The models used to fit the data have been described previously [24].Measurements were carried out in duplicate.

### 2.5. Synthesis of N-(3-methacrylamidopropyl)guanidinium Chloride

*N*-(3-Methacrylamidopropyl)guanidinium chloride was synthesized as previously described [25,26]. Briefly, a solution of *N*-(3-aminopropyl)methacrylamide HCl (15 g, 84 mmol), 4-methoxyphenol (150 mg) and *N*,*N*-diisopropylethylamine (38 mL, 209 mmol) in DMF (85 mL, keeping the final concentration of the reactants 2M), was stirred for 5 min under argon. To this solution *1H*-pyrazole-1-carboxamidine monohydrochloride (12.3 g, 84 mmol) was added. The mixture was stirred at room temperature for 24 h under Ar, then poured into diethylether (1200 mL). The resulting oil was separated from the supernatant and washed twice with a solution of acetonitrile (200 mL) and triethylamine (10 mL). The resultant solid was washed with dichloromethane (300 mL) and dried under vacuum to yield 13.3 g (72%) of the product (3, Supplemental Figure S1). ^1^H NMR (400 MHz, DMSO-d_6_) δ ppm 8.09(s, 1H), 7.91(s, 1H), 7.70–6.90(br, 4H), 5.70 (s, 1H), 5.33 (s, 1H), 3.16(m, 4H), 1.87 (s, 3H), 1.65 (quin, 2H).

### 2.6. Synthesis and Characterization of Poly(3-guanidinopropyl methacrylamide-co-acrylamide) HCl

*N*-(3-methacrylamidopropyl)guanidinium HCl (3.85 g, 17.4 mmol), acrylamide (0.64 g, 9 mmol), the RAFT agent 4-cyano-4-(thiobenzoylthio)pentanoic acid (0.063 g, 0.23 mmol), and the initiator azobisisobutyronitrile (7.4 mg, 0.045 mmol) were dissolved in dimethyl sulfoxide (DMSO) (25 mL) and degassed for 30 min. The solution was heated at 70 °C under argon for 40 h, then cooled and the copolymer precipitated in acetone. The precipitated copolymer was dissolved in methanol. Azobisisobutyronitrile (0.930 g, 5.7 mmol) was added, the solution was degassed for 30 min, and heated at 60 °C overnight to remove the RAFT agent. The solution was then cooled and the copolymer precipitated in acetone, filtered and dried. The final yield was 3.7 g or 83% (5, Supplemental Figure S1).

The mol% 3-guanidinopropyl sidechains was determined by ^1^H NMR (Supplmental Table S1). The M_m_ and PDI of the copolymers were determined by size exclusion HPLC using a CATSEC-300 column (Eprogen, Downers Grove, IL, USA) on an Agilent 1260 Infinity instrument. The copolymers were run in 1 wt % acetic acid and 0.10 M LiBr in HPLC grade H_2_O at a flow rate of 1 mL min^−1^ at 25 °C. The HPLC was equipped with a refractive index and miniDawn TREOS (Wyatt Technology, Santa Barbara, CA, USA) multi-angle light scattering detectors.

### 2.7. Preparation of Poly(3-guanidinopropyl methacrylamide-co-acrylamide) Sulfate

1 M Na_2_SO_4_ (9 mL) was added to an aqueous solution of poly(3-guanidinopropyl methacrylamide-*co*-acrylamide) HCl (500 mg) and stirred overnight. Two volumes of ethanol were added to precipitate the copolymer, which was then dissolved in H_2_O, and re-precipitated by the addition of 9 mL of 1M Na_2_SO_4_, followed by 2 volumes of ethanol. The precipitated copolymer was dissolved in H_2_O and passed through a column of IRA-400 anion exchange resin in sulfate form to ensure complete exchange of Cl^−^ with SO_4_^2−^ [27]. The solution was lyophilized, dissolved in water and refrigerated to separate the polymer from inorganic salts. Dissolving in water and refrigerating was repeated three times. The separated polymer was dissolved in water and lyophilized to obtain poly(3-guanidinopropyl methacrylamide-co-acrylamide) sulfate in a 69 % (360 mg) yield.

## 3. Results

### 3.1. Condensation of Salmine Sulfate

**Figure 1:**
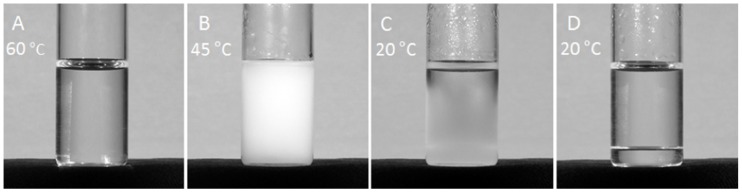
Temperature-dependent complex coacervation of salmine in 400 mM Na_2_SO_4_. (**A**) A solution of salmine sulfate (100 mg·mL^−1^) at 60 °C. (**B**) As the clear solution cooled, it became turbid at 45 °C when salmine sulfate condensed into complexes. (**C**) The complexes coalesced and spontaneously (1 g) settled out as a dense liquid precipitate. (**D**) After 24 h at 20 °C, the macrophase equilibrated into a clear homogeneous dense liquid.

The temperature dependent self-association of polycationic salmine sulfate into a condensed liquid phase is shown in Figure 1. A clear 100 mg mL^−1^ solution of salmine containing 400 mM Na_2_SO_4_ at 60 °C (Figure 1A) was placed in a 20 °C incubator to cool while the temperature of the solution was monitored. The solution abruptly turned cloudy at 45 °C, the upper critical solution temperature (UCST), as salmine sulfate associated into light scattering complexes (Figure 1B). The initial complexes condensed further into a cloudy liquid macrophase that spontaneously settled to the bottom of the bottle without centrifugation (Figure 1C). After 24 h at 20 °C, the condensed macrophase equilibrated into a transparent homogeneous liquid (Figure 1D, Supplemental Video S1).

### 3.2. Temperature (T) and Concentration Dependence of Salmine Sulfate Condensation

**Figure 2:**
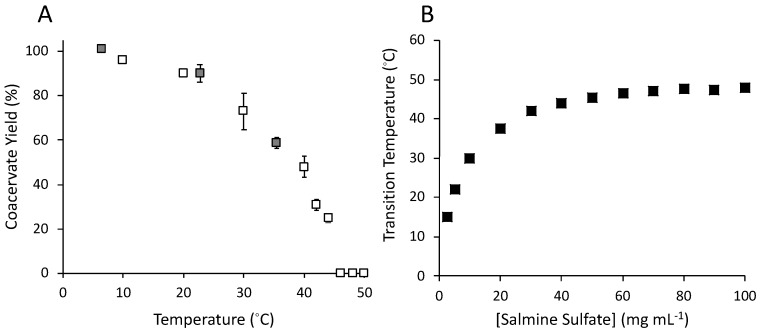
(**A**) Temperature dependence of condensed phase yield. Salmine = 50 mg·mL^−1^, sulfate (SO_4_^2−^) = 200 mM, R-Gdm^+^: SO_4_^2−^ = 1:1. Open symbols were determined by nuclear magnetic resonance (NMR). Grey squares were determined gravimetrically. Mean ± 1 SD (n=3). (**B**) Concentration dependence of the phase transition temperature with 1:1 molar ratios of positively charged guanidino (Gdm^+^) to SO_4_^2−^.

The percent of total salmine (50 mg·mL^−1^) in the condensed phase, referred to as yield, was determined as a function of T (Figure 2A). A 50 mg·mL^−1^ solution of salmine sulfate was estimated to comprise 194 mM Gdm^+^ sidechains and, as received, contained 99 mM SO_4_^2^^−^, corresponding to an approximately 0.5:1 molar ratio of SO_4_^2^^−^ to Gdm^+^ (see methods). Na_2_SO_4_ was added to a total of 200 mM for an approximate 1:1 molar ratio of Gdm^+^ to SO_4_^2^^−^. At 8 °C and below, nearly 100% of the salmine partitioned into the condensed phase. As T increased, the yield of condensed salmine decreased to 0% at 45 °C, corresponding to the phase transition temperature observed in Figure 2B. The salmine concentration dependence of the UCST—the cloud point temperature—was determined turbidimetrically (Figure 2B). The Gdm^+^ to SO_4_^2^^−^ molar ratio was adjusted to 1:1 to match the solution conditions of the temperature experiments. At 2.5 mg·mL^−1^, the UCST was 15 °C, and rose with increasing concentration until plateauing at 45 °C, at approximately 70 mg·mL^−1^. The UCST was not determined for salmine concentrations greater than 100 mg·mL^−1^.

### 3.3. Counter-Anion Dependence of Salmine Condensation

**Figure 3:**
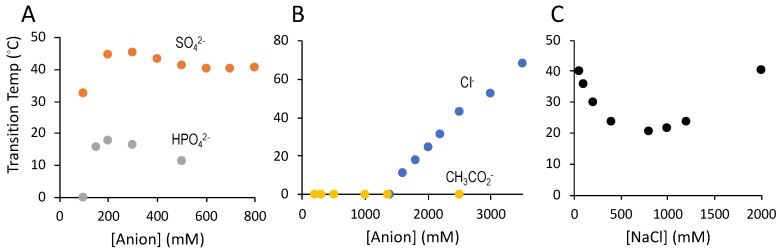
Counter-anion effects on salmine (50 mg·mL^−1^) upper critical solution temperature (UCST) determined by turbidimetry. (**A**) Dianions: Salmine sulfate (orange) and salmine phosphate dibasic (grey). (**B**) Monoanions: Salmine acetate (yellow) and salmine chloride (blue). (**C**) Effect of NaCl on the UCST of 50 mg·mL^−1^ salmine and 200 mM Na_2_SO_4_.

Sulfate concentration effects on salmine UCST were investigated by turbidimetry. The UCST of a 50 mg·mL^−1^ solution of salmine sulfate, as received without addition of Na_2_SO_4_, was 32 °C. Addition of Na_2_SO_4_ to a total of 200 mM, an approximate 1:1 molar ratio of Gdm^+^ to SO_4_^2−^, increased the UCST to 45 °C. Above 300 mM SO_4_^2−^, the UCST decreased slightly, then remained constant up to 800 mM SO_4_^2^^−^ (Figure 3A). The UCST of salmine exchanged with HPO_4_^2^^−^ was significantly lower than salmine sulfate, peaking at approximately 18 °C in 200 mM HPO_4_^2^^−^ at a 1:1 molar ratio. It was not possible to test HPO_4_^2^^−^ greater than 500 Mm, due to solubility limitations. Nevertheless, the limited data suggested that high [HPO_4_^2^^−^] suppressed the UCST more strongly than high [SO_4_^2^^−^].

The UCST is a direct measure of the stability of the intermolecular associations between salmine molecules that lead to condensation and phase separation. Based on the lower UCST, dianionic HPO_4_^2^^−^ promoted significantly weaker intermolecular associations between salmine molecules than SO_4_^2^^−^ dianions. The weak effect on UCST of high [SO_4_^2−^] suggested that excess SO_4_^2^^−^ ions do not competitively interfere with intermolecular associations that lead to condensation. On the other hand, excess HPO_4_^2^^−^ ions may competitively saturate Gdm^+^ sidechains preventing intermolecular ionic interactions.

The compositions of the condensed phases were determined at total SO_4_^2^^−^ concentrations of 99, 200, 400, and 800 mM (Table 1). There were no statistically significant differences in the condensed phases with respect to salmine concentration, water content, or the SO_4_^2−^ to Gdm^+^ molar ratio. The salmine concentration was greater than 600 mg·mL^−1^, representing a 12-fold spontaneous concentration of salmine into the condensed phase. The water concentration was approximately 50 wt % and the SO_4_^2−^ to Gdm^+^ molar ratio was approximately 0.5 at all [SO_4_^2−^]. The consistent molar ratio demonstrated that two positive Gdm^+^ sidechains were balanced by one SO_4_^2−^ dianion in the condensed phases, independent of the total [SO_4_^2−^]. The sulfate-mediated condensation is therefore due to specific intermolecular interactions, formation of stoichiometric complexes, rather than a solution effect or ‘salting out’ phenomenon. The constant condensed phase stoichiometry is consistent with the minimal effect of [SO_4_^2−^] on the UCST; excess [SO_4_^2−^] does not competitively disrupt the stoichiometric intermolecular binding of SO_4_^2−^ to Gdm^+^ sidechains that leads to condensation and associative macrophase separation.

Monovalent anions had dramatically different effects on salmine phase separation than divalent anions. Acetate ions did not promote LLPS at any concentration up to 2.5 M. Chloride ions, on the other hand, at concentrations of 1.4 M and higher induced LLPS with UCSTs that increased steadily with Cl^−^ concentrations up to 3.5 M (Figure 3B). The salmine concentration increased and water content decreased in the liquid coacervate phase as the NaCl increased (Table 1). It required about 10-fold higher concentrations of Cl^−^ than SO_4_^2^^−^ to induce LLPS. Addition of NaCl to salmine solutions with a 1:1 ratio of Gdm^+^ to SO_4_^2^^−^ suppressed UCST at low concentrations, then increased UCST at concentrations above 0.8–1 M (Figure 3C).

### 3.4. Isothermal Titration Calorimetry (ITC)

**Figure 4:**
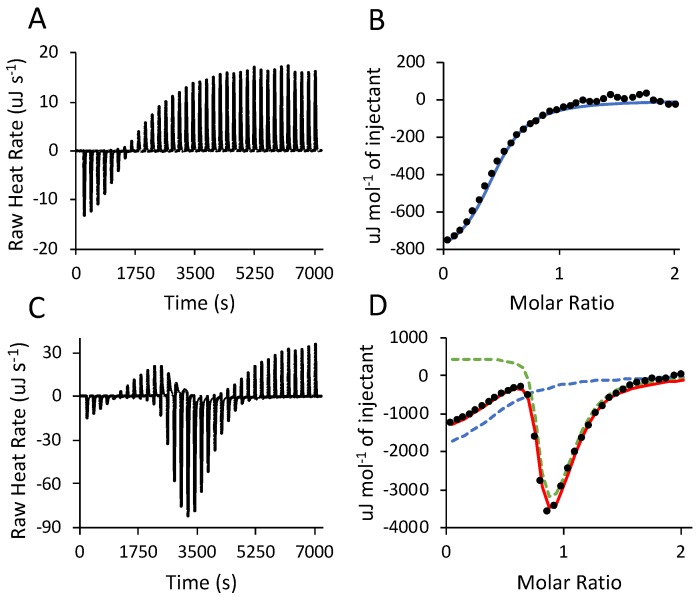
ITC thermograms of Na_2_SO_4_ titration into salmine chloride. (**A**) Raw heat flow rate per injection above the UCST at 25 °C. (**B**) A single-site binding model was fit (blue curve) to the integrated heat flow data (black symbols). (**C**) Raw heat flow rate per injection below the UCST at 10 °C. (**D**) The single-site (blue dashed curve) and a multiple independent site model (green dashed curve) were fit to the integrated heat flow data (black symbols). The red curve is the simultaneous fit of both models.

Heat flow associated with salmine sulfate condensation and phase separation were measured by isothermal titration calorimetry (ITC) to calculate the thermodynamic parameters of the reaction. A solution of salmine chloride (10 mg·mL^−1^, 41.4 mM Gdm^+^) was titrated with a solution of 400 mM Na_2_SO_4_ at 25 °C, above the UCST (Figure 4A). The thermograms from above the UCST show the heat flow due to sulfate ions binding to salmine chloride without the effects of heat flow due to intermolecular complexation and subsequent condensation of salmine sulfate. This control experiment enabled deconvolution and assignment of events to the titration data collected below the UCST. To our knowledge, there is no precedent literature wherein the effects of ionic interactions and counterion displacement were separated from subsequent condensation by titrating above and below the phase transition temperature. Most reports describing the thermodynamics of LLPS (complex coacervation) by ITC were done by titrating one polyelectrolyte (or polyampholyte) into a second oppositely charged polyelectrolyte at a temperature conducive to intermolecular complexation and phase separation (see ref. [27] for a recent review). Heat flow resulting from initial ionic interactions and counterion release were superimposed on heat flow from subsequent events during polyelectrolyte condensation.

Thermodynamic parameters were calculated for the 25 °C (above UCST) thermogram (Figure 4B) by fitting with a one-site model using software (Nanoanalyze^®^) supplied with the ITC instrument (Table 2, row 1). After subtracting the background heat of dilution of Na_2_SO_4_^2^^−^, the sulfate reaction with salmine chloride was exothermic, with a small enthalpic and a larger entropic contribution to the overall favorable change in free energy. The events contributing to the heat flow were likely the displacement of Cl^−^ counterions by SO_4_^2^^−^ ions, the enthalpically favorable formation of H-bonds between the SO_4_^2^^−^ oxygens and Gdm^+^ hydrogens, and the entahalpically disfavored release of H_2_O from the hydration shells of SO_4_^2^^−^ and Gdm^+^. The calculated 0.45 binding stoichiometry (N), a 1:2 molar ratio of SO_4_^2^^−^ to Gdm^+^, was consistent with the molar ratios determined by elemental analysis in the condensed phases (Table 1). The 25 °C ITC binding stoichiometry suggested that specific complexes are formed between two Gdm^+^ sidechains and one SO_4_^2^^−^ ion in the absence of macroscale condensation. In other words, pairing of Gdm^+^ sidechains by SO_4_^2^^−^ ions is intramolecular rather than intermolecular, above the UCST.

Below the UCST at 10 °C, three sequential events were apparent from inflections in the thermogram when salmine chloride (10 mg mL^−1^) was titrated with 400 mM Na_2_SO_4_ (Figure 4C). (The UCST at 10 mg mL^−1^ is lower than in Figure 2B because of the presence of Cl^−^.) The data were fitted simultaneously with both the one-site model (Figure 4D, blue dashed curve) and a multiple non-interacting sites (MNIS) model provided with Nanoanalyze^®^ (Figure 4D, green dashed curve). The calculated parameters from the one-site model were consistent with the parameters obtained for SO_4_^2^^−^ binding above the UCST; heat flow induced by interactions of SO_4_^2^^−^ with salmine chloride were exothermic, and driven both enthalpically and entropically (Table 2, row 2). There were small differences in the 10 °C ΔH and ΔS compared to 25 °C, because the temperature dependence of the heat capacity (ΔC_p_ = dH/dT) is not zero. However, the magnitude and stoichiometry provide confidence in the assignment of this event to the sulfate ions binding to salmine chloride.

The MNIS model separately identified additional events below the UCST which occurred after SO_4_^2^^−^ binding. The MNIS model treats macromolecules as having multiple non-cooperative binding sites, however it can also be interpreted as multiple events, rather than multiple binding sites [27,28]. Accordingly, the second sequential event was endothermic, overall enthalpically unfavorable, entropically favorable, and provided the largest driving force for LLPS (Table 2, row 3). We interpret this event as heat flow due to the initial condensation of salmine sulfate, corresponding to the sudden clouding of the solution at the UCST (Figure 1B). The comparatively large gain in entropy suggested the reaction was driven by hydrophobic effects, specifically restructuring and dehydration of the salmine molecules in the complexes. The third event, interpreted as the coalescence of condensed salmine sulfate complexes into a liquid macrophase, was enthalpically driven (Table 2, row 4). The MNIS model did not assume the events were sequential, but the approximately 1000-fold higher K_a_ associated with the second event suggests it occurred before the third, which is consistent with the inflections in the ITC data. The favorable enthalpy may be due to rearrangement between Gdm^+^ pairs into intermolecular bridges or clusters. The assumptions and multiple event interpretations are validated by the close fit to the data obtained by summing the binding isotherms from both the one-site and multi-site models (Figure 4D, red curve).

### 3.5. Condensation of Salmine Synthetic Analogs

Poly(3-guanidinopropyl methacrylamide-*co*-acrylamide) random copolymers were synthesized (Supplemental Figure S1) as salmine analogs to investigate whether the primary sequence, residues other than arginine, the chirality of the polypeptide backbone, or the salmine M_m_ monodisperisty were necessary, or contributed to LLPS. The first series, in which the guanidinopropyl sidechain concentration was varied from 10–65 mol %, the copolymers are referred to as pGPMAXX, where XX designates the target mol % of 3-guanidinopropyl sidechains. The target M_m_ of the RAFT polymerization reactions was 20 kg mol^−1^. In the second series, referred to as pGPMA65-MWXX, where XX designates the M_m_, the 3-guanidinopropyl was fixed at a target of 65 mol %, while the target M_m_ was varied from 10 to 40 kg mol^−1^ (Supplemental Table S1).

**Figure 5:**
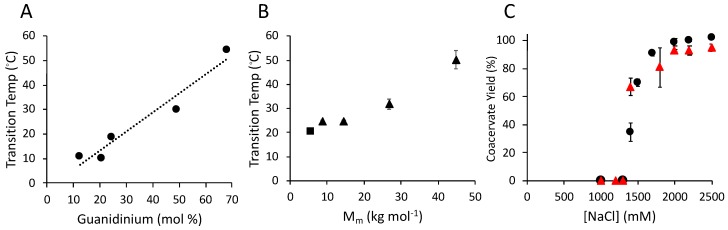
LLPS of pGPMA-*co*-acrylamide analogs of salmine determined by turbidimetry. (**A**) UCST dependence on mol % Gdm^+^. The copolymer concentrations were 50 mg·mL^−1^. The SO_4_^2^^−^:Gdm^+^ ratio was 1:1. The dashed line was added to guide the eye. (**B**) UCST dependence on copolymer M_m_. The copolymer concentrations (triangles) were 5 mg mL^−1^ and the SO_4_^2^^−^:Gdm^+^ ratio was 1:1. Square symbol = salmine sulfate at 5 mg mL^−1^ and the SO_4_^2^^−^:Gdm^+^ ratio was 1:1. Mean ± 1 SD (n = 3). (**C**) NaCl induced coacervation of pGPMA65 (triangles) and salmine (circles). Salmine 50 mg·mL^−1^ at 8 °C. Mean ± 1 SD (n = 3).

Upon cooling from above the UCST, the pGPMAXX sulfate copolymers first condensed into cloudy solutions, then spontaneously settled and equilibrated into transparent homogeneous liquid macrophases within 24 h (not shown), similar to salmine sulfate (Figure 1). At 10 mol% Gdm^+^, the lowest tested, pGPMA10 sulfate condensed at 10 °C. The UCST increased as the mol% of the guanidinopropyl sidechains increased due to increased stabilization of the condensed phase by the greater concentration of Gdm^+^/Gdm^+^ intermolecular interactions (Figure 5A). The UCST also depended on the copolymer M_m_ (Figure 5B). With the mol % Gdm^+^ approximately fixed, the UCST rose with increasing copolymer M_m_, due to a stabilization of the condensed phase by a greater number of intermolecular associations. Salmine sulfate (square symbol) was close to the copolymer M_m_ trendline. The pGMPMA65 copolymer, with a Gdm^+^ sidechain concentration similar to salmine, condensed at 55 °C, ten degrees higher than salmine sulfate due to the higher M_m_, 21.7 kg·mol^−1^ vs. 4.4 kg·mol^−1^. Chloride-induced LLPS of pGPMA65 (Figure 5C, circles) was also similar to salmine (Figure 5C, triangles), with LLPS beginning above 1.4 M NaCl and reaching almost 100% above 2 M.

The similar phase behavior of synthetic poly(Gdm^+^) copolymers to natural salmine demonstrated that intermolecular associations between Gdm^+^ groups alone are responsible for complexation and phase separation. The copolymers contain no negatively charged groups to participate in electrostatic interactions with the Gdm^+^ sidechains, nor aromatic groups to participate in π–cation interactions. The achiral backbone and statistical order of monomers demonstrated that neither the chiral polypeptide backbone, nor amino acid sequence of salmine, is necessary for LLPS of polyguanidiniums. A full comparison of the synthetic copolymers to salmine with regard to other ion effects and thermodynamics were beyond the scope of this initial report.

## 4. Discussion

### The Molecular Mechanism of Polyguanidinium LLPS

The absence of any negatively charged or aromatic residues in salmine or the pGPMA copolymers demonstrated condensation of poly(Gdm^+^)s was mediated strictly through Gdm^+^ sidechains in the presence of divalent anions, such as sulfate and phosphate. The consistent 0.5 molar ratio of SO_4_^2^^−^ to R-Gdm^+^ sidechains, whether calculated from ITC thermograms, or measured by elemental analysis of the condensed phase, demonstrated SO_4_^2^^−^ formed a specific chemical complex with pairs of Gdm^+^ groups. The energetics of SO_4_^2^^−^ binding to salmine had an overall favorable enthalpic contribution due perhaps to H-bond formation between SO_4_^2^^−^ oxygens and –NH_2_ of (R-Gdm^+^)_2_, and an overall favorable gain in entropy due likely to the displacement of Cl^−^ counterions and H-bonded H_2_O molecules. Hydrophobic effects due to stacking of poorly hydrated R-Gdm^+^ also likely contributes to the entropic driving force. Since the energetics were similar above and below the UCST, (Table 2) stoichiometric complex formation between SO_4_^2^^−^ and (R-Gdm^+^)_2_ does not lead directly to condensation and phase separation of salmine; the R-Gdm^+^ pairing must be largely intramolecular above the UCST (Figure 5A).

At and below the UCST, two additional transitons after SO_4_^2^^−^ (R-Gdm^+^)_2_ complexation were discerned by interpreting the data as arising from two sequential events. The first sharp transition created visible light scattering salmine complexes. This initial intermolecular association of salmine sulfate is endothermic, slightly unfavorable enthalpically, and entropically driven, likely due to dehydration and exclusion of water from the complexes. The second, slower transition results in the coalescence of the initial complexes into a homogenous dehydrated liquid network. 

The restructuring that occurs during the second transition is enthalpic, and may be driven by an exchange of intramolecular to intermolecular R-Gdm^+^ pairs (Figure 5B), or higher-order R-Gdm^+^ stacking, i.e., [Gdm^+^]_x_ clusters, as observed at the interface of multi-subunit proteins [29].

Condensation of R-rich proteins by high concentrations of Cl^−^ is likely not relevant to biological LLPS mechanisms, and a detailed comparison of Cl^−^ vs. SO_4_^2^^−^-induced LLPS was not a primary focus of this report. Nevertheless, chloride-induced LLPS of salmine was strikingly different than sulfate-induced LLPS; super-stochiometric concentrations were required to initiate phase separation, increasing Cl^−^ concentrations stabilized the condensed state, as reflected in the increasing UCST (Figure 3B), and progressively increased dehydration of the condensed phase (Table 1). In contrast, the extent of LLPS of oppositely charged polyelectrolytes steadily decreases as ionic interactions between macroions are screened by increasing [NaCl] [30,31]. The opposite effect of NaCl on salmine LLPS demonstrates that salmine self-association is not just carried out through simple electrostatic association of oppositely charged ions. Addition of low concentrations of NaCl to salmine sulfate decreased the stability of the salmine sulfate complexes, evident in the initial depression of the UCST, which then increased steadily above ~800 mM NaCl (Figure 3C). It appears SO_4_^2^^−^ and Cl^−^ promote salmine condensation by different mechanisms. Compared to divalent sulfate anions, higher concentrations of monovalent Cl^−^ may be required to sufficiently compensate or delocalize the positive charges of R-Gdm^+^ to allow pairing that leads to salmine condensation and dehydration. Or, the effect of Cl^−^ may be primarily on the solution—a salting out effect—enhanced by Gdm^+^ pairing. In this context, chloride is in the middle of the Hofmeister series with relatively low ability to salt out proteins.

**Figure 6:**
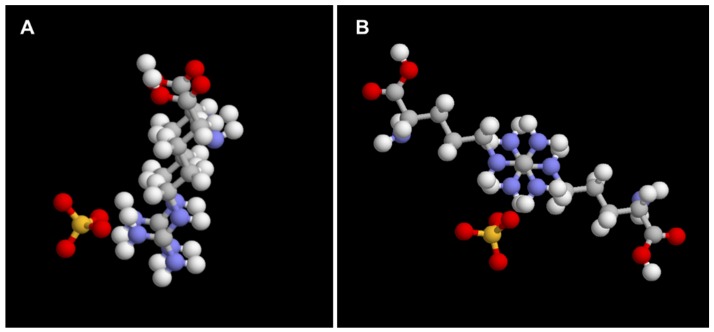
Parallel (**A**) and anti-parallel (**B**) stacking of R-Gdm^+^ sidechains in the presence of a sulfate molecule.

A molecular model of the proposed SO_4_^2^^−^ (Gdm^+^)_2_ stacking interactions, diagrammed in Figure 6, is derived from extensive evidence in the literature of R-Gdm^+^ contact pairing, the most compelling of which has been found in protein data bases [29,32]. A survey of the Brookhaven PDB by Scheraga’s group, identified numerous examples of R-Gdm^+^ pairs with less than 5 Å center-to-center separation of the R-Gdm^+^ groups in protein structures [32]. The Gdm^+^ pairs occurred as planar stacks equally distributed in two general orientations with respect to the long axis of the sidechain, parallel and anti-parallel. The stacks occurred in three rotational orientations with respect to the perpendicular axis of the Gdm^+^ pairs, referred to as eclipsed, staggered, and half-staggered. The stacked pairs were stabilized by H-bond bridges to H_2_O molecules and the Os of neighboring sidechains, in particular the carboxylates of glutamate and aspartate. Another PDB survey by Neves [29], identified over 70,000 examples of clustered Rs (<5 Å separation), occurring frequently at the interfaces of multi-subunit protein complexes, organized into pairs, strings with up to 7 members, and rings of 4–8 Rs. The R-Gdm^+^ groups were in stacked planar orientations, stabilized by H-bonds (5) with neighboring residues, water, or small anions.

In addition to the empirical evidence found in protein crystal structures, numerous MD simulations [33,34,35,36,37] have provided computational evidence of like-charge contact ion pairing between Gdm^+^, R-Gdm^+^, and oligo-R. Mason reported dynamic, nanometer-scale, network-like aggregations of Gdm^+^ ion pairs in the presence of sulfate with lifetimes on the order of several tenths of a nanosecond [38]. Similarly, Schneider et al. described H-bonded, worm-like chains of arginines in the presence of phosphate, citrate, and sulfate anions, but not in the presence of acetate or chloride anions [39]. Of the simulated anions, sulfate had the strongest interactions with the R-Gdm^+^ sidechains, consistent with the higher thermal stability (UCST) of the salmine sulfate complex coacervates we observed (Figure 3).

The counter-intuitive like-charge Gdm^+^ and R-Gdm^+^ stacked pairs are explained by the unique structure and chemistry of the guanidino functional group. The guanidinium ion, comprising three amino groups bonded to a central carbon atom, has been described as Y-conjugated and quasi-aromatic [40,41]. The rigid, planar geometry restricts H-bond donation by Gdm^+^ and alkyl-Gdm^+^ sidechains (six and five, respectively), and strong interactions with water, to the molecular plane of the ions. The delocalization of the π electron of the central C gives it low positive charge density and makes it a poor H-bond acceptor with H_2_O. As a result, the top and bottom surfaces of the ion are unhydrated. Gdm^+^ has been described as having essentially no hydration shell, and no effect on the structure or dynamics of water, based on neutron diffraction [42] and dielectric relaxation spectroscopy studies [43]. The main structural factors and forces that overcome electrostatic repulsion and stabilize the stacked Gdm^+^ pairs have been summarized by Vazdar and colleagues [44]. First, electrostatic repulsion is minimized by distribution of the positive charge between the three resonance structures of the planar molecules and staggering of the Gdm^+^ Ns by about 60° in the perpendicular axis of the stacked pair. Second, the weak electrostatic repulsion between the low charge density cations is overcome by energetically favorable dispersion forces due to (Gdm^+^)_2_ quadrapole-quadrapole interactions. Also, the excluded solvent volume, and associated energetic penalty, is lower for two or more stacked Gdm^+^ groups compared to single Gdm^+^, i.e., a hydrophobic driving force [33]. Additional stabilization is provided by H-bond bridging of (Gdm^+^)_2_ by water, neighboring residues, and small ions. Sulfate ions have the strongest stabilizing effect because of the highly favorable dimensions and tetrahedral geometry for bridging two Gdm^+^s [39].

Several of the proteins demonstrated to undergo LLPS in the cytoplasm and in vitro contain R-rich motifs, as well as aromatic residues (F, Y, and W) and acidic residues (D and E) [7,8,9,10,40,41]. In these cases, protein association has been attributed to electrostatic, π–cation, and π–π interactions. Like-charge (Gdm^+^)_x_ interactions within and between R-rich motifs were not anticipated. Favorable Gdm^+^ pairing in the presence of dianions is a mechanism in addition to electrostatic interactions that may contribute to LLPS. On the other hand, the R-rich repeating dipeptides deriving from GGGGCC hexanucleotide repeats associated with the gene C9orf72, contain only R, and no other charged or aromatic residues [18,19]. The C9orf72 hexanucletotide expansions are the primary genetic cause of familial amyotrophic lateral sclerosis and frontotemporal dementia [17]. The hexanucleotide repeats are translated from five of the six reading frames into repeating dipeptides, one of which encodes GR_n_ and another encodes PR_n_. The translated R-rich repeating dipeptides associate with nucleoli, disrupt RNA synthesis, and cause cell death [18]. Synthetic GR_20_ and PR_20_ polypeptides phase separate into liquid droplets in vitro in the presence of HPO_4_^2^^−^, as well as in the presence of 30% PEG and poly-U RNA, a polyanion [45]. In the absence of other charged or aromatic residues, as with salmine and synthetic poly(Gdm^+^), self-association of the toxic PR_n_ and GR_n_ must occur through R-Gdm^+^ pairing, although there may be other cellular factors involved.

## 5. Conclusions

The temperature-dependent condensation of salmine and synthetic polymer analogs into dense liquids is promoted by divalent dianions, such as sulfate and phosphate, and inhibited by monovalent anions such as chloride and acetate. The results provide empirical in vitro evidence of like-charge pairing between Gdm^+^ groups and highlight another potential mechanism of cellular LLPS. The sensitivity to environmental conditions of the salmine and synthetic poly(Gdm^+^) condensation and phase transitions provides an experimental approach for additional study of anion effects, thermodynamics, and the structure of like-charge Gdm^+^ ion pairing. The results strongly support the conclusions of others regarding the role of R-rich motifs in cellular LLPS, both regulated and pathological, providing additional details of the Gdm^+^ self-association mechanism, and the potent effect of specific anions to be considered in future studies of cellular LLPS. Detailed understanding of the mechanisms of specific anion promotion or disruption of R-mediated phase transitions may be useful during the search for small molecule inhibitors of pathological LLPS. Finally, the in vitro LLPS of salmine raises the question of whether protamine LLPS plays a role in the condensation of chromatin during spermatogenesis.

## Figures and Tables

**Table 1:** Composition of salmine coacervate phases.

Total [anion] (mM)	Salmine (mg mL^−1^)	Water (wt%)	Molar Ratio SO_4_^2−^ to Gdm^+^
**Salmine Sulfate**			
99	617 ± 33	49.8 ± 2.2	0.43 ± 0.03
200	634 ± 68	50.0 ± 4.0	0.44 ± 0.09
400	602 ± 11	47.8 ± 0.5	0.47 ± 0.00
800	643 ± 46	47.0 ± 1.5	0.47 ± 0.01
**Salmine Chloride**			
1400	465 ± 58	66.8 ± 4.3	--
1500	511 ± 11	60.9 ± 2.7	--
1700	553 ± 27	56.0 ± 0.8	--
2000	616 ± 17	55.3 ± 2.7	--
2200	626 ± 6	52.2 ± 1.4	--
2500	637 ± 6	48.5 ± 2.4	--

Mean ± s.d., n ≥ 3 for all measurements.

**Table 2:** ITC Thermodynamic Parameters.

Temperature (°K)	N	K_a_ (M^−1^)	H (kJ mol^−1^)	−TΔS (kJ mol^−1^)	G (kJ mol^−1^)
298.15 *	0.45	3.96 × 10^2^	−2.18	−12.65	−14.83
283.15	0.49	2.09 × 10^2^	−5.45	−7.13	−12.58
283.15	0.76	1.15 × 10^5^	1.15	−28.60	−27.45
283.15	0.33	4.90 × 10^2^	−11.71	−2.87	−14.58

* Above the phase transition temperature. N = binding stoichiometry.

## Supplementary Materials

The following are available online at https://www.mdpi.com/2073-4360/11/4/649/s1, Figure S1: Synthesis of poly(3-guanidinopropyl methacrylamide-co-acrylamide) HCl, Table S1: 3-guanidinopropyl methacrylamide copolymers, Video S1: Salmine condensation and phase separation.
