# Supplementary material for: Aqueous Liquid-Liquid Phase Separation of Natural and Synthetic Polyguanidiniums
Source: Polymers (Basel). 2019 Apr 9;11(4):649. doi: 10.3390/polym11040649 (PMC6523547; doi:10.3390/polym11040649)
Supplement: Supplementary file 1 [file polymers-11-00649-s001.zip › Supplemental Table S1.docx]

| **Table 3. 3-GuanidinoPropyl-MethAcrylamide Copolymers** | | | | |
| --- | --- | --- | --- | --- |
| Copolymer | Mol% Gdm^+^ | M_n_ (kg mol^-1^) | M_w_ (kg mol^-1^) | PDI |
| pGPMA10 | 12 | 16.3 | 19.2 | 1.18 |
| pGPMA20 | 21 | 17.0 | 21.1 | 1.24 |
| pGPMA25 | 25 | 19.5 | 22.9 | 1.17 |
| pGPMA50 | 49 | 14.5 | 16.7 | 1.15 |
| pGPMA65 | 68 | 21.7 | 25.8 | 1.19 |
| pGPMA-MW10 | 69 | 7.3 | 8.7 | 1.12 |
| pGPMA-MW15 | 67 | 12.6 | 14.4 | 1.14 |
| pGPMA-MW25 | 67 | 24.2 | 26.7 | 1.10 |
| pGPMA-MW40 | 72 | 42.3 | 44.8 | 1.06 |
