# Supplementary material for: Aqueous Liquid-Liquid Phase Separation of Natural and Synthetic Polyguanidiniums
Source: Polymers (Basel). 2019 Apr 9;11(4):649. doi: 10.3390/polym11040649 (PMC6523547; doi:10.3390/polym11040649)
Supplement: Supplementary file 1 [file polymers-11-00649-s001.zip › Supplemental Figure S1.docx]

**Figure S1.** Synthesis of poly(3-guanidinopropyl methacrylamide-co-acrylamide) HCl.
